# Successful external validation of a model to predict other cause mortality in localized prostate cancer

**DOI:** 10.1186/s12916-016-0572-z

**Published:** 2016-02-09

**Authors:** Matthew Kent, David F. Penson, Peter C. Albertsen, Michael Goodman, Ann S. Hamilton, Janet L. Stanford, Antoinette M. Stroup, Behfar Ehdaie, Peter T. Scardino, Andrew J. Vickers

**Affiliations:** Department of Epidemiology and Biostatistics, Health Outcomes Research Group, Memorial Sloan-Kettering Cancer Center, 485 Lexington Avenue, 2nd Floor, New York, NY 10017 USA; Department of Surgery, Urology Service, Memorial Sloan Kettering Cancer Center, New York, NY USA; Department of Urologic Surgery, Vanderbilt University, Nashville, TN USA; Geriatric Research Education and Clinical Center, VA Tennessee Valley Healthcare System, Nashville, TN USA; Division of Urology, University of Connecticut Health Center, Farmington, CT USA; Department of Epidemiology, Emory University, Atlanta, GA USA; Department of Preventive Medicine, Keck School of Medicine of the University of Southern California, Los Angeles, CA USA; Division of Public Health Sciences, Fred Hutchinson Cancer Research Center, Seattle, WA USA; New Jersey State Cancer Registry, Trenton, NJ USA

**Keywords:** Life expectancy, Clinical decision support, Prediction, Radical prostatectomy

## Abstract

**Background:**

Although life expectancy estimation is vital to decision making for localized prostate cancer, there are few, if any, valid and usable tools. Our goal was to create and validate a prediction model for other cause mortality in localized prostate cancer patients that could aid clinician’s initial treatment decisions at the point of care.

**Methods:**

We combined an adjusted Social Security Administration table with a subset of comorbidities from a UK actuarial life expectancy model. Life tables were adjusted on the basis of survival data from a cohort of almost 10,000 radical prostatectomy patients treated at four major US academic institutions. Comorbidity-specific odds ratios were calculated and incorporated with baseline risk of mortality. We externally validated the model on 2898 patients from the Prostate Cancer Outcomes Study, which included men diagnosed with prostate cancer in six SEER cancer registries. These men had sufficient follow-up for our endpoints of 10- and 15-year mortality and also had self-reported comorbidity data.

**Results:**

Life expectancy for prostate cancer patients were close to that of a typical US man who was 3 years younger. On external validation, 10- and 15-year concordance indexes were 0.724 and 0.726, respectively. Our model exhibited excellent calibration. Taking into account differences between how comorbidities are used in the model versus how they were recorded in the validation cohort, calibration would improve for most patients, but there would be overestimation of the risk of death in the oldest and sickest patients.

**Conclusions:**

We successfully created and externally validated a new life expectancy prediction model that, while imperfect, has clear advantages to any alternative. We urge consideration of its use in counseling patients with localized prostate cancer.

**Electronic supplementary material:**

The online version of this article (doi:10.1186/s12916-016-0572-z) contains supplementary material, which is available to authorized users.

## Background

Life expectancy plays an important role when determining appropriate treatment options for prostate cancer patients. The slow growing nature of the disease complicates treatment decisions because some patients, such as those who are older or who have significant comorbidity, should not be candidates for invasive procedures such as radical prostatectomy. The National Comprehensive Cancer Network (NCCN) recommends active surveillance or observation as an alternative treatment option for managing prostate cancer in patients who meet specific guidelines based on tumor characteristics and life expectancy. However, the guidelines make questionable recommendations on how clinicians should estimate life expectancy for prostate cancer patients [1]. The current advice is to adjust the Social Security Administration tables [2] by adding 50 % for patients in the top quartile and subtracting 50 % for patients in the lowest quartile of health. This is a problematic approach for two reasons. First, the determination of a patient’s health quartile is left up to the subjective judgment of the clinician, an approach that is not validated. Second, the method results in some unusual recommendations, such as surgery being an option for 25 % of 80-year-olds with low risk cancer: life expectancy for an 80-year-old is 8 years; add 50 % for patients in the top quartile of health gives 12 years; and surgery is included as a possibility for patients with a life expectancy of 10 years or more.

The American Urological Association (AUA) guidelines similarly recommend different treatment options based on patient life expectancy: “Life expectancy, rather than patient age, is a major factor to consider in treatment selection” [3]. Although the AUA mentions life expectancy throughout their guidelines, and states that patients with shorter life expectancies may opt for active surveillance, the only reference for estimating life expectancy is the 2003 United States life tables [4].

As life expectancy estimation is integrated directly with the clinical recommendations of major guidelines, one might expect a plethora of tools to be available in the literature for clinician use. We previously reported a systematic review in which we found a dearth of accurate life expectancy tools that could be incorporated into clinical workflow [5, 6]. Tools gave estimates of life expectancy that could not be integrated with cancer risk estimates, used invalid or unsubstantiated methods to adjust life expectancy for comorbidity, and provided estimates that appear implausible or could not be easily used in routine clinical practice. For example, the most widely used comorbidity index, Charlson [7], gives HIV a risk score 6 times greater than that of myocardial infarction. In a contemporary cohort of patients presenting with prostate cancer, it is unclear if these are reasonable risk estimates. As another example, Kim et al. published an online tool to estimate prostate cancer mortality [8]. For a typical contemporary patient with screen-detected cancer, the model uses only age and Gleason score and cannot incorporate health status. Moreover, risks seem on the high side: a 65-year-old male of average health and Gleason 6 disease is given an 11 % risk of prostate cancer mortality, despite there being many investigators who question whether Gleason 6 even counts as cancer [9]. Given the importance of using life expectancy to determine treatment options for prostate cancer patients, and the lack of validated life expectancy estimation tools available to assist clinicians, we aimed to build a prediction model for the risk of death from other causes based on patient age and comorbidities. Specifically, the model would provide risk estimates and not remaining number of life years, use a valid approach to defining comorbidities, be in a form that could be readily implemented in the clinical setting, provide plausible risk estimates, and could be individualized to each patient.

## Methods

The typical approach for building a prediction model—obtaining a data set with outcomes and predictors and then fitting coefficients—is not optimal for estimating comorbidity-adjusted risk of non-cancer mortality in patients presenting with prostate cancer. This is primarily because even the largest prostate cancer data sets are too small to provide accurate estimation for the effects of each of a wide range of comorbidities. Therefore, we chose to take an alternative approach, creating the model by combining information from a variety of different sources.

We started with the measure of actuarial life expectancy (MALE) model to estimate comorbidity risk [10]. This model was selected on the grounds that it had been developed using a very large life insurance database and was the only model we found to provide the risk of death from specific comorbidities. However, we found the MALE model far too complex to be used in routine clinical practice due to the level of detail required for each comorbidity, such as the classification of angina or the sub-type of atrial fibrillation. Our approach therefore was to use a reduced subset of MALE comorbidities, chosen in consultation with urologists at Memorial Sloan Kettering Cancer Center (MSKCC). The comorbidities included hypertension (defined as systolic ≥160 mm Hg and diastolic ≥90 mm Hg), angina, congestive heart failure, heart attack, aortic stenosis, atrial fibrillation, asthma, abdominal aortic aneurysm, peripheral vascular disease, deep venous thrombosis, pulmonary embolus, stroke, smoking, and diabetes. In some cases, the MALE questions were simplified down, for instance, the original MALE question on pulmonary embolus gave different risks for recurrent vs. single episode; this was modified to ask only about any history of pulmonary embolus. We also included cholesterol in the model because some comorbidities had different risk estimates for different cholesterol levels. These levels include high total cholesterol (>270 mg/dL) and low high-density lipoprotein (HDL) (<20 mg/dL).

The MALE model only provided risks of death for each comorbidity at 10 and 15 years. To convert these risks into odds ratios, we took the ratio of risk of death with no comorbidities to the risk of death with the comorbidity of interest. This allowed us to calculate odds ratios at both 10 and 15 years. If a patient presented with multiple comorbidities, their odds ratios were multiplied together.

Next, we estimated baseline risk of death on the basis of age. We found the MALE model to be poorly calibrated for the US population. For instance, the model gives an estimated 15-year survival probability of 55 % for a healthy 65-year-old. The estimate for all 65-year-old Americans from the Social Security Administration life tables [2] is higher, at 59 %, even though this estimate includes men who are seriously ill. We therefore chose to recalibrate the MALE model on the basis of US data, but with an adjustment factor to reflect the population that presents for treatment of localized prostate cancer is healthier than average. To derive this adjustment, we analyzed survival data from close to 10,000 radical prostatectomy patients treated at four major US academic institutions [11], data for which has been published. We explored the difference between survival curves in our data set and those of the Social Security Administration tables. Once a patient’s age-specific baseline risk of death was obtained it could be adjusted accordingly based on their comorbidities to obtain a final estimate of comorbidity-adjusted risk of death.

We then externally validated our final model on a cohort of 2,898 patients from the Prostate Cancer Outcomes Study (PCOS) [12]. This cohort consisted of men diagnosed with biopsy-proven prostate cancer between October 1, 1994 and October 31, 1995 in six of the SEER cancer registries in the states of Connecticut, Utah, and New Mexico and the Metropolitan areas of Atlanta (GA), Los Angeles (CA), and Seattle (WA). These men had sufficient follow-up for our endpoints of interest and also had information on a subset of the comorbidities used in our model, including angina, congestive heart failure, diabetes, heart attack, stroke, and high blood pressure. These comorbidities were captured using a self-reported health-related quality of life (HRQOL) questionnaire at 6, 12, and 24 months, which allowed patients to indicate if they were ever diagnosed with a specific comorbidity. However, this cohort did lack some of the comorbidities used in our model, specifically aortic stenosis, atrial fibrillation, asthma, abdominal aortic aneurysm, peripheral vascular disease, deep venous thrombosis, pulmonary embolus, smoking, and cholesterol levels. Furthermore, hypertension was defined more liberally in PCOS than is used in our model. Hence we planned to interpret our results in the context of differences in comorbidity documentation. Discrimination, as measured by the Harrell’s concordance index, and calibration were used to assess model fit at both 10 and 15 years. Our model was “locked down” before being applied to the validation cohort, and no changes were made subsequently. All statistical analyses were performed using Stata 12.0 (StataCorp, College Station, TX, USA).

## Results

When estimating baseline risk of death, we found close to a 3-year difference in survival between radical prostatectomy patients and the general population represented by the Social Security Administration tables. In other words, the survival curves for death from other causes seen amongst 60-year-olds presenting for prostate cancer treatment approximately mirrored those seen in 57-year-olds in the Social Security Administration data. Therefore, we shifted the age-associated risks from the Social Security Administration table down by 3 years to obtain the risks used in our model. Table 1 presents a subset of these risks at both 10 and 15 years.

**Table 1:** Risk of overall death at 10 and 15 years by age

Age (years)	10 years		15 years	
	SSA risk	SSA-adjusted risk	SSA risk	SSA-adjusted risk
50	8 %	6 %	14 %	11 %
51	8 %	7 %	15 %	12 %
52	9 %	7 %	16 %	13 %
53	10 %	8 %	17 %	14 %
54	10 %	8 %	19 %	15 %
55	11 %	9 %	20 %	16 %
56	12 %	10 %	21 %	17 %
57	13 %	10 %	23 %	19 %
58	14 %	11 %	25 %	20 %
59	15 %	12 %	26 %	21 %
60	16 %	13 %	28 %	23 %
61	17 %	14 %	31 %	25 %
62	18 %	15 %	33 %	26 %
63	20 %	16 %	35 %	28 %
64	22 %	17 %	38 %	31 %
65	23 %	18 %	41 %	33 %
66	25 %	20 %	44 %	35 %
67	27 %	22 %	47 %	38 %
68	30 %	23 %	51 %	41 %
69	32 %	25 %	54 %	44 %
70	35 %	27 %	58 %	47 %
71	37 %	30 %	61 %	51 %
72	40 %	32 %	65 %	54 %
73	43 %	35 %	69 %	58 %
74	47 %	37 %	73 %	61 %
75	50 %	40 %	77 %	65 %

SSA risk comes from the Social Security Administration risk tables, while the adjusted risk takes into account a 3-year age shift due to patients presenting with localized prostate cancer being healthier on average. The latter estimates are used in our model

The 10- and 15-year odds ratios for the subset of comorbidities from the MALE model are presented in Table 2. Our subset consisted of 14 different comorbidities with varying levels of risk within comorbidities and also interactions between comorbidities. For example, diabetes has multiple odds ratios depending on how long a patient has been a diabetic. Similarly, some comorbidities are influenced by cholesterol, with different odds ratios for different cholesterol levels. These interactions and variations in risk were derived directly from the MALE model.

**Table 2:** Comorbidity odds ratios for 10- and 15-year risk of death

Comorbidity	10 years	15 years
Hypertension	1.29	1.38
Angina	1.55	1.62
+ High total cholesterol	1.84	2.08
+ Low HDL	2.26	2.72
+ High total and low HDL	3.12	4.09
Congestive heart failure	3.82	3.67
Heart attack	1.55	1.62
+ High total cholesterol	1.84	2.08
+ Extremely low HDL	2.26	2.72
+ High total cholesterol and low HDL	3.12	4.09
Aortic stenosis	1.29	1.38
+ High total cholesterol	1.62	1.76
+ Extremely low HDL	2.00	2.27
+ High total cholesterol and low HDL	2.77	3.67
Atrial fibrillation	1.29	1.38
Asthma (mild)	1.17	1.17
Asthma (moderate)	2.00	2.27
Asthma (severe)	2.45	2.85
Abdominal aortic aneurysm	1.92	1.99
Peripheral vascular disease	1.62	1.76
+ High total cholesterol	2.00	2.27
+ Extremely low HDL	2.36	2.85
+ High total and extremely low HDL	3.25	4.60
Deep venous thrombosis	1.84	1.99
+ High total cholesterol	2.17	2.60
+ Extremely low HDL	2.56	3.30
+ High total and extremely low HDL	3.52	4.89
Pulmonary embolus	1.00	1.00
+ High total cholesterol	1.29	1.38
+ Extremely low HDL	1.62	1.76
+ High total and extremely low HDL	2.36	2.85
Current smoker	2.00	2.00
Former smoker	1.50	1.50
Diabetes		
0–5 years	1.00	1.00
6–10 years	1.62	1.76
11–20 years	2.00	2.27
>20 years	2.36	2.85
Stroke		
Hemorrhage	1.62	1.76
Infarction, thrombosis, embolism	2.36	2.85

For example, a 65-year-old man would have a risk of death from other causes at 10 years of 18 % (Table 1), an odds of 18:82. If he smoked (odds ratio of 2.0), this would shift the odds to 36:82, a risk of 36 ÷ (36 + 82) = 31 % risk. Similar calculations could be made to calculate the man’s risk if he had angina (odds ratio of 1.55, risk 25 %) or both angina and smoking (odds ratio of 1.55 × 2.0, risk of 40 %)

**Fig. 1:**
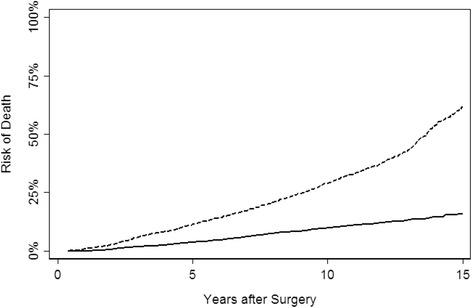
Cumulative hazard curves for the PCOS cohort. Solid line, prostate cancer; dashed line, other cause mortality

Table 3 presents the patient characteristics of the PCOS cohort used for model validation. As shown, the cohort included data on only a subset of the necessary comorbidities included in our model. In total, 1,041 patients died from other causes and 307 died from prostate cancer with a median follow-up time for survivors of 13 years. Figure 1 shows the probability of prostate cancer survival; 10- and 15-year survival was 91 % and 85 %, respectively.

**Table 3:** PCOS validation set patient characteristics. All values are median (IQR) or frequency (%)

Characteristic	N = 2,898
Patient age	67 (60, 73)
Patient race	
White	2,032 (70 %)
Black	475 (16 %)
Hispanic	391 (13 %)
Patient treatment type	
Surgery	1,448 (50 %)
Radiation	688 (24 %)
Watchful waiting	483 (17 %)
Isolated androgen deprivation therapy (ADT)	279 (10 %)
Angina	387 (13 %)
Congestive heart failure	210 (7.2 %)
Diabetes	512 (18 %)
Heart attack	305 (11 %)
High blood pressure	1,234 (43 %)
Stroke	146 (5.0 %)

**Fig. 2:**
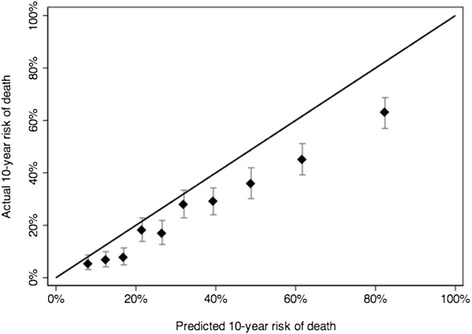
10-year calibration plot

**Fig. 3:**
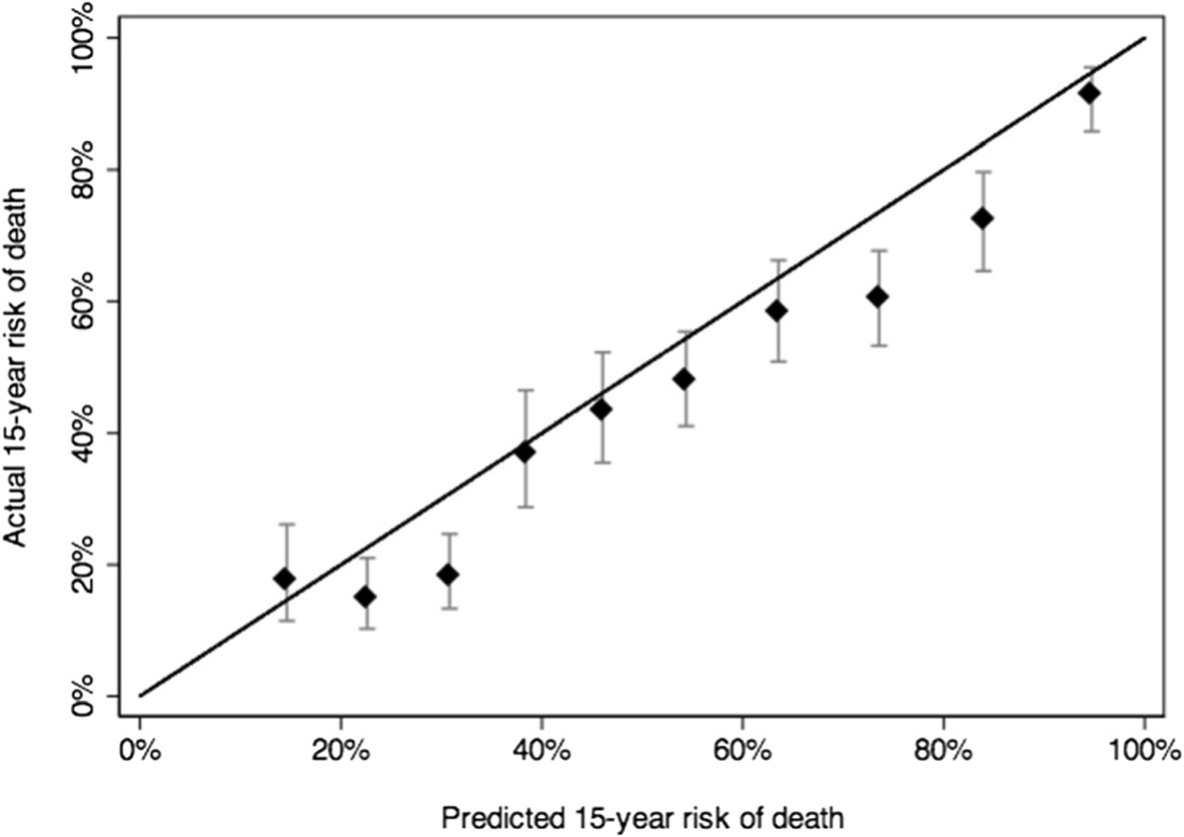
15-year calibration plot

When we applied our model to the PCOS cohort we found the discrimination (c-index) to be 0.724 and 0.726 at 10 and 15 years, respectively. Figures 2 and 3 present the 10- and 15-year calibration plots. We can see that our model exhibited generally good calibration for 10-year risk of death, but overestimated risk of death for patients at very high risk (>50 % risk of death at 10 years). For 15-year risk of death, we found much less miscalibration. Additionally, it can be seen from the calibration plots, the models separate risk very well. For example, in both the 10- and 15-year plots, 10 % of patients have risks of death less than 15 % and 10 % have risks greater than 90 %.

## Discussion

We previously found deficiencies in current tools that predict life expectancy or risk of death from other causes. Tools were either difficult to use in the clinical setting or provided questionable estimates or recommendations, such as surgery for low risk cancer in a healthy 80-year-old [5]. We created a new model by making common sense adjustments to two existing tools. We reduced risks obtained from US life tables on the grounds that these include patients too ill to present for treatment of localized prostate cancer, and adapted a UK-based comorbidity assessment model to make it feasible to use clinically. We applied this new model to an independent cohort and found that our model exhibited good discrimination and calibration.

Our model is not without limitations. It is not ideal to have to merge an adaptation of a UK-based comorbidity assessment with modified US life expectancy data. But we had little choice: data sets with long-term follow-up of localized prostate cancer have not had rich information on comorbidities nor been large enough to estimate the prognostic impact of these comorbidities.

Additionally, the PCOS study did not capture all the comorbidities used in our model. Therefore, since some comorbidities were unreported, the patient’s risk of death would have been underestimated in the current study, compared to using the model in practice when the full spectrum of comorbidities would be obtained. However, comorbidities reported in PCOS are likely correlated with the unreported comorbidities; for instance, atrial fibrillation (unreported) with history of heart attack (reported)—this would shift the highest risk patients to the right on the calibration curve. On the other hand, PCOS used a lower cut-point for hypertension than in our model, with the result that more PCOS patients than expected had hypertension. Correcting for this effect would shift the calibration curve to the left, leading to an improved estimate of performance overall. Hence the anticipated effect of inconsistency between the PCOS comorbidities and those used in our model would be improved calibration for most patients, but overestimated risk for the oldest and sickest patients. We believe that such overestimation is clinically irrelevant, because whether a patient has, say, a 90 % or 95 % risk of death from other causes at 15 years would make little or no difference to clinical decision making.

Our tool can be combined with existing untreated prostate cancer mortality estimates to obtain an adjusted risk of prostate cancer mortality, taking into account age and comorbidities. Rider et al. [13] present risks of untreated prostate cancer mortality classified into risk and age categories. These estimates were from 117,328 Swedish men who were not treated with curative intent. However, since the cohort consisted of patients from 1991 to 2009, we had to take into account the shift in Gleason grading that took place in the early 2000s [14]. We assumed that 25 % of patients previously scored as Gleason 6 in 1990–2005 would be Gleason 7 on contemporary grading. Upgraded patients were assigned a risk equal to the average of the low and intermediate groups. These estimates are based on a study in which tumors graded in 1990–1992 were regraded in 2002–2004: the net upgrading from Gleason 6 was 19 %, with about one-third of upgrades to Gleason 8 or higher; this is approximately equivalent in risk terms to 25 % being upgraded to Gleason 7 [15]. The adjusted risks are presented in Additional file 1: Table S1. To determine a patient’s risk, Rider et al. grouped patients by clinical stage, Gleason score, and prostate-specific antigen (PSA). Although PSA is an extremely strong predictor of mortality in the population of men without a cancer diagnosis [16–18], it is not a strong predictor of mortality in men who have been diagnosed with localized prostate cancer, once stage and grade are known [19]. Accordingly, we exclude PSA as a risk criterion, that is, assignment to high, low, or intermediate risk is based purely on stage and grade.

Given the risks from our life expectancy model and the adjusted estimates for untreated prostate cancer mortality, we are able to calculate using conditional probability a key estimate of clinical value: the probability that the patient would die of prostate cancer were he to be managed conservatively, taking into account the probability that he would not die of another cause. Additional file 1: Table S2 shows example data and outputs for a selection of patients. Note that this includes patients with a risk of other cause mortality greater than 50 % at 10 years, that is, patients with a life expectancy less than 10 years: there is no reason why the model cannot be used with such patients.

These models are currently available on https://www.mskcc.org/nomograms/prostate under male life expectancy. Additional file 1: Figure S1 shows a report of the results that displays the text describing both the patient’s comorbidities and cancer characteristics; the risk of death from untreated prostate cancer; comorbidity-adjusted risk of death; and risk of death from prostate cancer, taking into account comorbidities. We intend to refine further the model for prostate cancer death to take into account differences in risk within Gleason categories (e.g. 3 + 4 vs. 4 + 3) as well as the different impact of stage compared to grade.

We are aware that our model is limited by the predefined set of comorbidities. Naturally, it would not be feasible to include all of the many thousands of diseases, and their subcategorizations, in a tool to be used in clinic. There would also be considerable computational challenges in modeling how the prognostic effect of many diseases changes over time, as new treatments are developed. Hence, as would be true for any model, clinical judgment needs to be exercised when using the estimates from our model to aid treatment decision making. Such clinical judgment would need to incorporate considerations of disease severity (for instance, early stage congestive heart failure compared to disabling disease); diet, exercise and other lifestyle factors; and comorbidities not included in the tool (consider a patient recently diagnosed with advanced colorectal cancer).

## Conclusions

Estimating the risk of other cause mortality is a key aspect of treatment decision making in early stage prostate cancer. We have previously demonstrated the need for improved tools to make this assessment. As such, we urge consideration of our model for counseling prostate cancer patients about treatment options.

## Availability of data and materials

Data are available on request to the authors.

## Additional file

Additional file 1: Table S1. 10- and 15-year prostate cancer mortality by age and risk group. Low risk, stage T1–T2, and Gleason score ≤6; intermediate risk, stage T1–T2, and Gleason score 7); high risk, stage ≥ T3 OR Gleason score ≥8. **Table S2.** Model inputs and outputs for some example patients. Risk of death from prostate cancer from the Swedish data; risk of death from other causes from the MALE model applied to US life tables; risk of death from prostate cancer, taking into account death from other causes. **Figure S1.** Life expectancy report. (DOCX 195 kb)
