# Supplementary material for: Successful external validation of a model to predict other cause mortality in localized prostate cancer
Source: BMC Med. 2016 Feb 9;14:25. doi: 10.1186/s12916-016-0572-z (PMC4748497; doi:10.1186/s12916-016-0572-z)

**Appendix Table 1. 10 and 15-year prostate cancer mortality by age and risk group.** Low risk: Stage T1-T2 & Gleason score ≤6; intermediate risk: Stage T1-T2 & Gleason score 7); high risk: Stage ≥ T3 OR Gleason score ≥ 8

| **Risk Group** | **Age** | **10-Year Probability of Death** | **15-Year  Probability of Death** |
| --- | --- | --- | --- |
| Low | <65 Years | < 1% | 4% |
| Low | 65-75 Years | 3% | 8% |
| Low | >75 Years | 6% | 9% |
| Intermediate | <65 Years | 8% | 16% |
| Intermediate | 65-75 Years | 11% | 19% |
| Intermediate | >75 Years | 14% | 19% |
| High | <65 Years | 31% | 40% |
| High | 65-75 Years | 28% | 39% |
| High | >75 Years | 29% | 33% |

**Appendix Table 2. Model inputs and outputs for some example patients**. Risk of death from prostate cancer from the Swedish data; risk of death from other causes from the MALE model applied to US life tables; risk of death from prostate cancer taking into account death from other causes.

| Patient | | | Death from prostate cancer | | Death from other causes | | Death from prostate cancer taking into account death from other causes | |
| --- | --- | --- | --- | --- | --- | --- | --- | --- |
| Age | Comorbidities | Prostate cancer risk | 10 years | 15 years | 10 years | 15 years | 10 years | 15 years |
| 60 | Smoker | Intermediate | 8% | 16% | 24% | 39% | 6% | 11% |
| 72 | Former smoker, angina, high cholesterol | Intermediate | 11% | 19% | 58% | 80% | 5% | 6% |
| 50 | Diabetes > 20 years, hypertension, heart attack | Low | < 1 % | 4% | 25% | 46% | < 1% | 2% |
| 58 | Mild asthma | High | 31% | 40% | 13% | 23% | 27% | 34% |
| 72 | Peripheral vascular disease, high cholesterol | High | 28% | 39% | 50% | 74% | 14% | 17% |
| 55 | None | Low | < 1% | 4% | 9% | 16% | < 1% | 3% |
| 65 | None | Low | 3% | 8% | 19% | 33% | 2% | 6% |
| 76 | None | Low | 6% | 9% | 43% | 69% | 3% | 4% |
| 55 | None | Intermediate | 8% | 16% | 9% | 16% | 7% | 14% |
| 65 | None | Intermediate | 11% | 19% | 19% | 33% | 9% | 14% |
| 76 | None | Intermediate | 14% | 19% | 43% | 69% | 8% | 10% |
| 55 | None | High | 31% | 40% | 9% | 16% | 28% | 36% |
| 65 | None | High | 28% | 39% | 19% | 33% | 23% | 30% |
| 76 | None | High | 29% | 33% | 43% | 69% | 17% | 18% |

**Appendix** **Figure 1.** Life expectancy report.


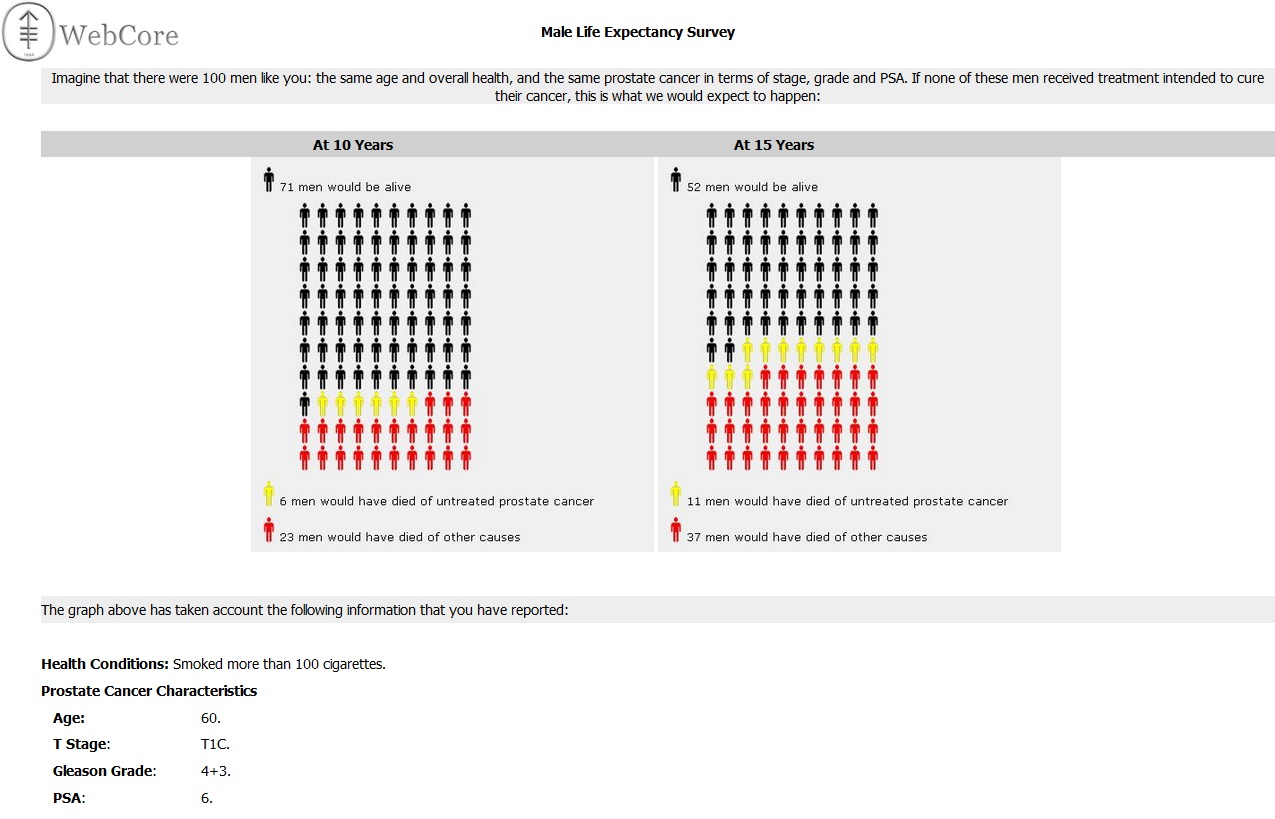

Supplement: Additional file 1: Table S1. — 10- and 15-year prostate cancer mortality by age and risk group. Low risk, stage T1–T2, and Gleason score ≤6; intermediate risk, stage T1–T2, and Gleason score 7); high risk, stage ≥ T3 OR Gleason score ≥8. Table S2. Model inputs and outputs for some example patients. Risk of death from prostate cancer from the Swedish data; risk of death from other causes from the MALE model applied to US life tables; risk of death from prostate cancer, taking into account death from other causes. Figure S1. Life expectancy report. (DOCX 195 kb) [file 12916_2016_572_MOESM1_ESM.docx]
